# Does antithrombotic prophylaxis worsen early outcomes of total thyroidectomy? - a retrospective cohort study

**DOI:** 10.1186/s12893-018-0407-2

**Published:** 2019-04-24

**Authors:** E. Erdas, F. Medas, S. Sanna, L. Gordini, G. Pisano, G. L. Canu, P. G. Calò

**Affiliations:** Department of Surgical Sciences, University of Cagliari, Cittadella Universitaria, SS554, Bivio Sestu, 09042 Monserrato, CA Italy

**Keywords:** Antithrombotic prophylaxis, Thyroid surgery, Postoperative bleeding

## Abstract

**Background:**

Currently, there is no strong evidence on the effectiveness and safety of pharmacological antithrombotic prophylaxis in thyroid surgery. The aim of this study was to establish whether the prophylactic use of low-molecular-weight heparin (LMWH) could negatively affect the early outcomes of patients undergoing total thyroidectomy.

**Methods:**

Data from patients submitted to total thyroidectomy between February 2013 and October 2017 were retrospectively collected and analysed. Only patients with indication to antithrombotic prophylaxis according to current guidelines were included in the study. Eligible cases were divided into two groups, which corresponded to two distinct periods of our surgical practice: Group A, which included 178 consecutive patients who were submitted to antithrombotic prophylaxis with LMWH, and Group B, which included 348 consecutive patients who did not receive prophylaxis. Primary endpoints were the incidence of post-operative cervical haematomas (POCH) and thromboembolic events. Secondary endpoint was the length of postoperative hospital stay. Statistical analysis was performed by using Student’s t test for continuous variables and Chi-square test for categorical variables. A *P* value of less than 0.05 was considered statistically significant.

**Results:**

The two groups of patients were comparable in terms of age, gender, thyroid disease, duration of surgery, and weight of the thyroid gland. Overall, no thromboembolic events were registered. The comparative analysis of the other outcome measures, showed no significant differences between the two groups (POCH: 2 cases (1.12%) in Group A vs 8 cases (2.30%) in Group B - *p 0.349*; Postoperative hospital stay: 2.90 ± 0.86 days in Group A vs 2.89 ± 0.99 days in Group B - *p 0.908*).

**Conclusions:**

Data from this study do not support or contraindicate the use of antithrombotic prophylaxis in thyroid surgery. However, since thyroidectomy is a closed-space procedure, and even modest bleeding may quickly result in airway compression and death by asphyxia, mechanical prophylaxis should be preferred to LMWH whenever possible.

**Trial registration:**

ISRCTN ISRCTN12029395. Registered 05/02/2018 retrospectively registered.

## Background

Post-operative cervical haematoma (POCH) is a rare but potentially lethal complication of thyroid surgery. In major centers it occurs in 0.3–1.6% of patients, thus representing only 8% of all complications [1]. Although mortality after thyroidectomy is extremely low (0.065%), it is often related to the occurrence of POCH, since bleeding into the deep neck space can quickly result in airway compression and death by asphyxia [2, 3]. Thus, post-operative bleeding is one of the major concerns of surgeons after thyroidectomy, and most of their efforts are addressed in its prevention [4, 5].

POCH is generally considered an unpredictable complication of thyroid surgery, however, in recent years, it has been shown that anticoagulant drugs significantly increase the risk of perioperative bleeding, which can also occur several days after surgery [6–8]. More specifically, Lloyd et al. showed that, even when anticoagulants are administered at low doses in order to prevent deep vein thrombosis (DVT), they may increase the risk of post-operative bleeding by 0,5% [9].

According to the American College of Chest Physicians (ACCP) guidelines, DVT prophylaxis with low-molecular-weight heparin (LMWH) should be administered to all patients over 40-year-old undergoing surgical procedures at low risk of bleeding, which last longer than 45 min [10]. Although ACCP guidelines make no specific reference to the type of surgery, these recommendations can be applied to thyroid surgery and, for this reason, have been entirely transposed into the position statement of the Italian Association of Endocrine Surgery Units (UEC Club), recently incorporated in the newly founded United Italian Society of Endocrine Surgery (SIUEC) [11]. Even before its definitive publication in June 2016, many Italian endocrine surgery units, including ours, started to apply these recommendations. However, currently there is no evidence that DVT prophylaxis is useful and safe in thyroid surgery, as it has been shown for other major procedures.

The aim of this retrospective cohort study was to establish whether the introduction of this new protocol in our surgical practice has negatively affected the early outcome of patients undergoing total thyroidectomy, with particular reference to the incidence of POCH and duration of post-operative hospital stay.

## Methods

### Study design and patient selection

This is a retrospective cohort study of 526 patients submitted to total thyroidectomy at the Endocrine Surgery Unit of the University Hospital in Cagliari, Italy, between February 2013 and October 2017. Data were extracted from the institutional computer-based register of thyroid surgery. All patients submitted to total thyroidectomy for whom antithrombotic prophylaxis would be indicated according to ACCP guidelines were included in the study. The exclusion criteria were age below 18 years, anticoagulant or antiplatelet therapy, recurrent goitre and operations other than total thyroidectomy (hemithyroidectomy, thyroidectomy with central or lateral neck dissection). Eligible cases were divided into two groups: Group A, which included 178 consecutive patients treated in the period between June 2015 and October 2017 who were submitted to antithrombotic prophylaxis with LMWH according to the ACCP Guidelines; Group B, which included 348 consecutive patients operated on before this period, within the same timeframe (28 months), who did not receive prophylaxis. Mechanical prophylaxis with elastic stockings or intermittent pneumatic compression has never been used in our series.

For each group, the following variables were analysed: age, gender, indication for surgery, length of the operation, weight of the thyroid gland, length of postoperative hospital stay, and early postoperative complications (within one month after surgery).

All the patients provided written informed consent for the storage and use of their data.

### Clinical endpoints

The primary outcome measures were the incidence of POCH and thromboembolic events. POCH was considered as postoperative bleeding requiring surgical revision.

The secondary outcome measure was the length of postoperative hospital stay. This data was used as a surrogate for the amount and progress (continuing, increasing, decreasing) of drains collection, which is the main limiting factor for an early discharge in our unit.

Finally, although not strictly useful for the purposes of this study, the rate of recurrent laryngeal nerve injury, hypocalcaemia and wound infection was also recorded.

All patients underwent telephone follow-up once a week for at least one month.

### Statistical analysis

Data were analysed by means of IBM SPSS 22 software. Statistical analysis was performed by using Student’s t test for continuous variables and Chi-square test for categorical variables.

A *P* value of less than 0.05 was considered statistically significant.

### Preoperative care

#### Antibiotic prophylaxis

Second generation cephalosporin is administered only in selected cases, such as patients affected by diabetes mellitus, immunosuppression and obesity.

#### Antithrombotic prophylaxis

From June 2015, ACCP guidelines for the prevention of DVT in non-orthopaedic surgical patients have been introduced in our surgical practice. Thus, a subcutaneous prophylactic dose (i.e., 0.3 ml) of nadroparin calcium is administered the day before the operation to all patients over 40 year-old, and continued for at least 10 days.

#### Management of hyperthyroidism

All patients affected by hyperthyroidism are preventively treated with antithyroid drugs in order to restore euthyroidism prior to surgery.

Patients awaiting surgery for Graves’ disease received 10 days of Lugol’s solution pre-operatively (0.5 ml 3 times daily).

### Surgical technique

Operations in both groups were performed by experienced endocrine surgeons, who followed a standardised technique of total extracapsular thyroidectomy.

Recurrent laryngeal nerves and parathyroids were systematically searched and identified. Intraoperative nerve monitoring (IONM) was routinely used in order to facilitate nerve identification and verify its functional integrity. Haemostasis was mainly achieved by FOCUS harmonic scalpel (Ethicon Endo-Surgery, Inc., Cincinnati, Ohio, USA), although major vessels were also tied proximally with an absorbable ligature. In all patients, two closed suction drains were placed below the strap muscles, to be removed when the secretion volume was less than 20 ml over 24 h. The strap muscles and platysma were reaproximated by interrupted 3/0 Vicryl and the skin incision was sutured using intradermal 3/0 polypropylene.

### Postoperative care

All patients are subject to a close monitoring in the first 12 h after surgery in order to detect early signs of cervical haematoma, respiratory distress or hypocalcaemia. Drains are removed when the daily amount of fluid collection falls below 20 ml in each reservoir.

Parathyroid hormone (PTH) and serum calcium levels are measured on the first and second postoperative day, while further controls are performed only if required (PTH at very low level, clinical signs of hypocalcaemia regardless of serum calcium levels). Post-operative oral calcium and vitamin D supplements are administered in all symptomatic patients with hypocalcaemia.

After discharge, all patients were re-evaluated within 1 week in order to assess the wound and, if appropriate, remove intradermal suture.

## Results

The two groups of patients were comparable in terms of age, gender, thyroid disease, duration of surgery, and weight of the thyroid gland (Table 1). In both groups, no general complications, as respiratory, cardiologic or thromboembolic events were registered. Overall, the average length of the postoperative hospital stay was less than 3 days and the incidence of postoperative complications was in line with the literature data. The comparative analysis of all outcome measures, showed no significant differences between the two groups (POCH: 2 cases (1.12%) in Group A vs 8 cases (2.30%) in Group B - *p 0.349*; Postoperative hospital stay: 2.90 ± 0.86 days in Group A vs 2.89 ± 0.99 days in Group B - *p 0.908*) (Table 2).

**Table 1:** Demographic and clinical features

	Group A (*n* = 178)	Group B (*n* = 348)	*P value*
Gender			
- Male	42 (23.60%)	88 (25.29%)	*0.670*
- Female	136 (76.40%)	260 (74.71%)	
Age (mean ± SD)	60.08 ± 7.87	60.31 ± 8.93	*0.771*
Length of the operation (min) (mean ± SD)	92.10 ± 23.36	94.78 ± 26.90	*0.259*
Histological diagnosis			
- Malignancy	54 (30.34%)	126 (36.21%)	*0.179*
- Benign disease	124 (69.66%)	222 (63.79%)	
Thyroid weight (g) (mean ± SD)	48.91 ± 47.79	41.98 ± 34.30	*0.056*

Abbreviations: *min: minutes; g: grams*

**Table 2:** Comparative analyses of clinical outcomes

	Group A (*n* = 178)	Group B (*n* = 348)	*P value*
Postoperative hospital stay (d) (mean ± SD)	2.90 ± 0.86	2.89 ± 0.99	*0.908*
Hypoparathyroidism	61 (34.27%)	113 (32.47%)	*0.678*
RLN injury	3 (1.69%)	7 (2.01%)	*0.796*
POCH	2 (1.12%)	8 (2.30%)	*0.349*
Wound infection	1 (0.56%)	–	*0.163*

Abbreviations*: d: days; SD: standard deviation; RLN: Recurrent laryngeal nerve; POCH: Post-operative cervical haematoma*

## Discussion

Currently, there are no specific guidelines for the use of DVT prophylaxis in thyroid surgery. Indeed, the recommendations reported in the positional statement of UEC Club is a merely transposition of ACCP guidelines concerning the prevention of venous thromboembolism in non-orthopaedic surgical patients [10, 11]. Unfortunately, these guidelines are based on studies that did not test the effectiveness and safety of DVT prophylaxis in thyroid surgery, which differs from most of the other surgical procedures for some specific peculiarities. Although the risk of post-operative bleeding is low (about 1% in major centers) [1], thyroidectomy should be included among the so-called closed-space procedures, such as intracranial neurosurgery and spinal canal surgery, for which even minor bleeding may result in life-threatening complications [10]. It has been reported that only 1% of all post-operative bleedings requires blood transfusions, which means 0.12% of all thyroidectomies [1]. However, since the deep cervical space is a closed compartment, even small bleedings can quickly result in airway compression and death by asphyxia [3]. For these reasons, according to the above-mentioned guidelines, mechanical prophylaxis, such as intermittent pneumatic compression, should be preferred to LMWH in thyroid surgery [10].

The question that some authors tried to answer is whether the reduction of the venous thromboembolism rate expected from the pharmacological prophylaxis is favourable in terms of risk/benefit ratio in thyroid surgery. In this regard, Roy et al. [12] showed that after thyroid and parathyroid surgery the risk of bleeding requiring a return to the operating room was 1.58%, which is 10-fold greater than the risk of developing a DVT. The authors concluded that DVT prophylaxis should be evaluated at the discretion of the surgeon in select high-risk patients. More recently, Docimo L et al. [13] reached similar conclusions after analysing the outcomes of 1018 consecutive thyroidectomies performed over a period of 6 years. In this study, the rate of DVT in patients undergoing total thyroidectomy without preoperative prophylaxis is 8-fold less than the rate of postoperative bleeding (0.1% vs 0.8%). The authors concluded that DVT prophylaxis should be given on a case-by-case basis, according to the individual patient risk factors.

Another topic that has been recently discussed refers to the potential role of thyroid cancer in promoting DVT in patients undergoing thyroidectomy. It has been reported that patients affected by various malignant tumours have a four- to seven-fold increased risk of developing venous thromboembolism, and almost 10% of ambulatory cancer patients dies from thromboembolic events [14, 15]. However, Reike CE et al. [16] registered a very low risk of DVT, without significant differences between malignant (0.08%) and benign disease (0.07%), among 19,640 patients undergoing thyroidectomy over a period of 4 years. The authors concluded that the need for DVT prophylaxis should be determined by individual patient risk factors, regardless of the nature of thyroid disease. They also argued that the low rates of DVT observed in their study, even in the highest risk patients, demonstrated the need for further studies to determine which patients undergoing thyroidectomy might benefit from DVT prophylaxis.

The aim of the present study was to determine whether the ACCP guidelines on antithrombotic prophylaxis were applicable to thyroid surgery. For this purpose we compared the early outcomes of patients undergoing total thyroidectomy before and after the introduction of these guidelines in our surgical practice. We expected that the incidence of POCH and the length of hospital stay would be higher during the period in which antithrombotic prophylaxis was administered. However, we did not find any significant differences between the two periods. Furthermore, we did not record any case of venous thromboembolism. Thus, the present study did not demonstrate the usefulness of antithrombotic prophylaxis in reducing the risk of thromboembolism in thyroid surgery. At the same time, the application of ACCP guidelines did not worsen the post-operative outcomes in terms of haemorrhagic risk and length of hospital stay. However, this study has several limitations, such as the small patient population and its retrospective nature; thus its results should be considered with caution. Further larger, multicenter, randomised studies are needed to enhance the evidence on this topic.

## Conclusions

Data from this study and the review of the Literature do not support or contraindicate the use of antithrombotic prophylaxis in thyroid surgery.

Currently, there is no evidence that LMWH significantly reduces the incidence of DVT or increases the incidence of POCH and the length of hospital stay. However, it should be emphasised that thyroidectomy is a closed-space procedure, and even modest bleedings may result in airway compression and death by asphyxia. For this kind of procedures, ACCP guidelines recommend the use of mechanical prophylaxis, preferably with intermittent pneumatic compression. In patients at high or very high risk of DVT, mechanical prophylaxis should be continued until the risk of bleeding decreases and pharmacologic prophylaxis may be initiated.

## Abbreviations

ACCP: American College of Chest Physicians
DVT: Deep vein thrombosis
LMWH: Low-molecular-weight heparin
POCH: Post-operative cervical haematoma
PTH: Parathyroid hormone
RLN: Recurrent laryngeal nerve
SIUEC: United Italian Society of Endocrine Surgery
UEC Club: Italian Association of Endocrine Surgery Units
